# The effect of continuous intercostal nerve block vs. single shot on analgesic outcomes and hospital stays in minimally invasive direct coronary artery bypass surgery: a retrospective cohort study

**DOI:** 10.1186/s12871-022-01607-7

**Published:** 2022-03-08

**Authors:** Youxiu Yao, Mao Xu

**Affiliations:** Department of Anesthesiology, Peking University Third Hospital, Beijing, People’s Republic of China

**Keywords:** Minimally invasive surgery, Coronary artery bypass, Intercostal nerve, Block, Outcomes, Length of stay

## Abstract

**Background:**

Minimally invasive direct coronary artery bypass (MIDCAB) grafting surgery is accompanied by severe pain. Although continuous intercostal nerve block (CINB) has become one of the multimodal analgesic techniques in single port thoracoscopic surgery, its effects on MIDCAB are unclear. The purpose of this study was to compare the effects of CINB and single shot on analgesic outcomes and hospital stays in patients undergoing MIDCAB in a real-world setting.

**Methods:**

A retrospective cohort study was carried out at Peking University Third Hospital, China. Two hundred and sixteen patients undergoing MIDCAB were divided into two groups: a CINB group and a single block (SI) group. The primary outcome was postoperative maximal visual analog scale (VAS); secondary outcomes included the number of patients with maximal VAS ≤ 3, the demand for and consumed doses of pethidine and tramadol, and the length of intensive care unit (ICU) and hospital stays. The above data and the area under the VAS curve in the 70 h after extubation for the two subgroups (No. of grafts = 1) were also compared.

**Results:**

The maximum VAS was lower in the CINB group, and there were more cases with maximum VAS ≤ 3 in the CINB group: CINB 52 (40%) vs. SI 17 (20%), *P* = 0.002. The percentage of cases requiring tramadol and pethidine was less in CINB, *P* = 0.001. Among all patients, drug doses were significantly lower in the CINB group [tramadol: CINB 0 (0–100) mg vs. SI 100 (0–225) mg, *P* = 0.0001; pethidine: CINB 0 (0–25) mg vs. SI 25 (0–50) mg, *P* = 0.0004]. Further subgroup analysis showed that the area under the VAS curve in CINB was smaller: 28.05 in CINB vs. 30.41 in SI, *P* = 0.002. Finally, the length of ICU stay was shorter in CINB than in SI: 20.5 (11.3–26.0) h vs. 22.0 (19.0–45.0) h, *P* = 0.011.

**Conclusions:**

CINB is associated with decreased demand for rescue analgesics and shorter length of ICU stay when compared to single shot intercostal nerve block. Additional randomized controlled trial (RCT) is needed to support these findings.

**Supplementary Information:**

The online version contains supplementary material available at 10.1186/s12871-022-01607-7.

## Background

Minimally invasive direct coronary artery bypass (MIDCAB) grafting surgery avoids median sternotomy [1] by means of a 6 ~ 7 cm long incision located in the left chest that avoids damage to either ribs or sternum, and is widely favored for its advantages such as minimal invasiveness, lower blood transfusion requirements, fast recovery and more satisfactory appearance [2–5]. However, this anterolateral thoracotomy will be accompanied by severe and sometimes uncontrollable pain, which may develop into chronic pain in the future. Chronic post thoracotomy pain is common and is associated with acute severe postoperative pain in 30–50% of patients [6]. The nociceptive stimulation induced by the pain will cause the release of catecholamines, resulting in disturbances in patients' breathing, metabolism, endocrine [7] and immune responses [8], which will increase hospital stays and costs. Although high dose opioids such as morphine can be used for postoperative intravenous analgesia, their effects are not ideal, and can also cause the inhibition of respiration and hemodynamics [8]. In order to achieve precise analgesic effects and facilitate postoperative recovery, the use of regional blocks such as epidurals or paravertebral blocks is possible [9, 10], but these are complex and carry a risk of related complications, which are not applicable in the case of anticoagulants, hemodynamic instability, vertebral fracture, etc. Intercostal nerve block, due to its ease of administration and safety [11, 12] has become one of the multimodal analgesic techniques in single port thoracoscopic surgery [13]; however, the effects of continuous intercostal nerve block (CINB) on MIDCAB are unclear. Since CINB is a promising first-line interventional analgesic technique routinely used for MIDCAB in our institution, the purpose of this study is to retrospectively analyze the analgesic outcomes of this technique in the real world and its impact on hospital stays.

## Methods

### Study design and participants

**Fig. 1:**
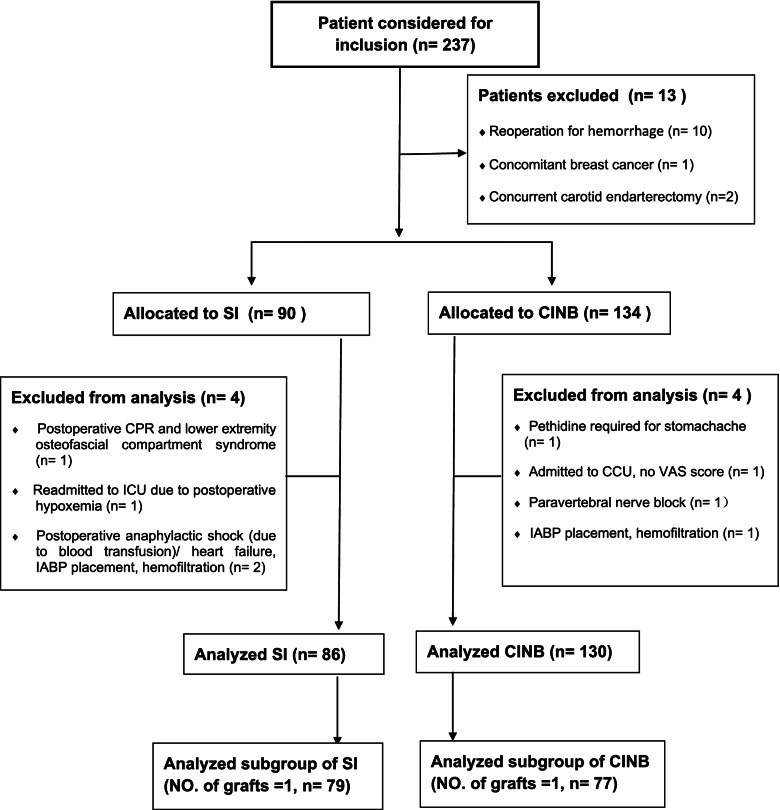
Flow chart of study. SI: single injection, CINB: continuous intercostal nerve block, CPR: cardiopulmonary resuscitation, ICU: intensive care unit, IABP: intra-aortic balloon pump, CCU: cardiac care unit, VAS: visual analogue scale

This retrospective cohort study was approved by the Ethics Committee of Peking University Third Hospital. Electronic medical records were retrieved retrospectively. The study included patients undergoing MIDCAB in Peking University Third Hospital from June 2014 to August 2017, who were given SI or CINB as the main part of their pain management program. Primary exclusion criteria included: reoperation for hemorrhage, concomitant breast cancer, and concurrent carotid endarterectomy. Once the enrolled population was established, the remaining data were retrieved through medical records and noted in case report forms for further analysis. The secondary exclusion criteria for data analysis included: postoperative cardiopulmonary resuscitation, readmission to ICU, anaphylactic shock / heart failure, IABP placement, hemofiltration, pain beyond the surgical site such as toothache or stomachache requiring rescue analgesics, other regional blocks with overlapping effects, and unavailable key data such as VAS score. Further subgroup analysis was conducted for patients with No. of grafts = 1 in the two groups. A flow chart of the study is shown in Fig. 1.

### CINB and SI administration

For the control group (SI), a heart surgeon experienced in the use of intercostal nerve block administered extrapleural intercostal nerve block at the fourth intercostal space (incision space) of the left anterior axillary line before closing the thoracic incision at the conclusion of surgery. 0.5% ropivacaine 20 ml was injected through an epidural Tuohy needle without catheterization, and 10 ml was infiltrated in the upper and lower adjacent intercostals respectively. For the CINB group, on the basis of the SI scheme, a thin flexible epidural catheter was threaded 3–4 cm beyond the needle tip, and properly fixed by suturing; 250 ml of 0.2% ropivacaine was infused using an ambulatory PCA (patient-controlled analgesia) pump; device controls were set to 5 ml/h continuous infusion, 5 ml/press on demand, lockout time 15 min.

### Postoperative analgesia protocol

The postoperative analgesia protocol in this study was as follows: For both groups, butorphanol 1 mg q. 6 h was administered intravenously before removal of the tracheal tube. ICU or ward nurses scored the patients using a VAS after extubation and noted the responses in the medical records. Supplemental doses of rescue tramadol and/or pethidine were given according to the degree of pain. SI group patients received 100 mg tramadol orally starting from 4 ≤ VAS < 6, and 25 mg rescue pethidine was injected intramuscularly when VAS ≥ 6 as directed by the doctor. CINB group patients received 0.2% ropivacaine firstly by pressing a PCA pump, assisted by the nurse, and then followed the scheme for SI patients in the situation when their VAS score remained ≥ 4 after 20 min’ observation.

### Outcome measures

The primary outcome of interest is the maximal VAS score (patients self-rated their subjective pain intensity by means of a visual analog scale graded from “no pain” = 0 to “the most unbearable pain” = 10).

Secondary outcomes included the number of patients with maximal VAS score ≤ 3, the cases of supplemental demand for and consumed doses of pethidine and tramadol, postoperative intubation time, length of ICU and hospital stay, postoperative untoward effects, e.g., nausea and vomiting, somnolence, skin pruritus or dyspnea, and pulmonary complications visible in a chest X-ray (atelectasis, pulmonary exudation, pleural effusion and pneumothorax).

### Subgroup analysis

Since a greater number of grafts would tend to be accompanied by longer duration of surgery and more surgical trauma, it was necessary to restrict subgroup analysis to single graft cases. In this way, the similarities in trauma degree and duration of surgery make for greater comparability between the two groups and for more convincing research regarding the attendant pain. The relevant data for the two subgroups (No. of grafts = 1) was analyzed. In addition, since VAS scores can only be self-rated after extubation, the area under the VAS score curve, SpO_2_, respiratory rate, systolic blood pressure, diastolic blood pressure and heart rate of the two subgroups in 10 time periods in the 70 h after extubation (T1: 0-2 h, T2: 4-6 h, T3: 10-12 h, T4: 20-22 h, T5: 26-28 h, T6: 34-36 h, T7: 44-46 h, T8: 50-52 h, T9: 58-60 h, T10: 68-70 h) were recorded, analyzed and compared.

### Statistical analysis

For both primary and secondary outcomes, Kolmogorov–Smirnov tests for normality were performed. Student’s t-tests (parametric) or Mann–Whitney/Wilcoxon rank-sum tests (non-parametric) were conducted to compare continuous variables data; categorical data were analyzed using the Chi square or Fisher exact tests. Analysis of the outcomes for sub-group patients was also performed based on whether patients received continuous or single injection intercostal nerve blocks. ANOVA with repeated measures was used to compare the data between the two subgroups at consecutive different time points. Time to event curves were constructed using the Kaplan–Meier method, with *P* < 0.05 defined as significant. All data management and analyses were executed in GRAPHPAD PRISM 6 statistical software.

## Results

### Patient recruitment and numbers analysis

224 patients were recruited after primary exclusion (reoperation for hemorrhage/*n* = 10, concomitant breast cancer/*n* = 1, concurrent carotid endarterectomy/*n* = 2), with 90 being allocated to SI and 134 to CINB (Fig. 1). Secondary exclusions were performed in further analysis after retrieval of patient medical records, including 4 cases in each group. The four patients excluded from the SI group were: postoperative CPR and lower extremity osteofascial compartment syndrome (*n* = 1); readmission to ICU due to postoperative hypoxemia (*n* = 1); postoperative anaphylactic shock due to blood transfusion/heart failure, IABP placement, hemofiltration (*n* = 2). The four patients excluded from the CINB group were: pethidine required for stomachache (*n* = 1); admitted to CCU (cardiac care unit), no VAS score (*n* = 1); paravertebral nerve block (*n* = 1); IABP placement, hemofiltration (*n* = 1). Ultimately, 86 patients in SI and 130 in CINB were analyzed. Further subgroup analysis was performed for patients with No. grafts = 1 in the two groups (SI group/*n* = 79, CINB group/*n* = 77) (Fig. 1).

### Baseline characteristics and operative details

Patient demographics and operative characteristics are presented in Table 1. The demographics, comorbidities, and cardiopulmonary function data were similar for the two groups. The number of patients with No. grafts ≥ 2 in the CINB group was more than that in the SI group (*P* < 0.0001), and the median duration of surgery was 0.4 h longer for patients in the CINB group: CINB 2.9 (2.1–4.6) h vs. SI 2.5 (2.1–3.0) h, *P* = 0.003. (Table 1).

**Table 1:** Patient demography, Comorbidities, and Operative Data

Parameters	Group CINB (*n* = 130)	Group SI (*n* = 86)	*P*
Age, years	62.0 ± 9.5	62.6 ± 10.6	0.665
Weight, kg	69.2 ± 11.6	69.6 ± 10.0	0.809
Male, %	98(75%)	59(69%)	0.280
BMI, kg.m^−2^	25.0 ± 3.3	25.0 ± 2.9	0.995
Hypertension	89 (68%)	54 (63%)	0.388
Diabetes	41 (32%)	26 (30%)	0.839
NYHA			0.814
I/asymptomatic	42(32%)	30(35%)	
II	65(50%)	45(52%)	
III	21(16%)	10(12%)	
IV	2(2%)	1(1%)	
Pulmonary function ^a^			
FVC actual, L	3.31 ± 0.82	3.12 ± 0.86	0.130
FVC% predicted	89 ± 14	85 ± 15	0.114
FEV_1_ actual, L	2.49 ± 0.72	2.45 ± 0.68	0.767
FEV_1_% predicted	85 ± 18	86 ± 16	0.630
Pre. PO_2_, mmHg^#^	82 ± 18	81 ± 15	0.742
Pre. PCO_2_, mmHg ^#^	39 ± 4	40 ± 4	0.647
Pre. glucose, mmol/L	7.8(5.9–9.6)	7.0(5.8–9.2)	0.164
No. of grafts			< 0.0001
1	77(59%)	79(92%)	
2	45(35%)	7(8%)	
3	8(6%)	/	
Duration of surgery, h	2.9(2.1–4.6)	2.5(2.1–3.0)	0.003

Values are mean ± SD, median (interquartile range), or number with percentage

*Abbreviations*: *SI* Single injection, *CINB* Continuous intercostal nerve block, *NYHA* New York Heart Association, *FEV*_*1*_ Forced expiratory volume in 1 s, *FVC* Functional vital capacity, *BMI* Body mass index, *Pre* Preoperative

^a^Indicates that only 77 patients in CINB group had preoperative pulmonary function data. # Indicates *n* = 82 in SI, and *n* = 123 in CINB

### Primary and secondary outcomes

The maximum VAS score of the CINB group was lower than that of the SI group: CINB 5.00 (3.00–5.00) vs. SI 6.00 (4.75–6.00), *P* < 0.001. Additionally, there were significantly more cases with maximum VAS score ≤ 3 in the CINB group: CINB 52 (40%) vs. SI 17 (20%), *P* = 0.002. (Table 2).

**Table 2:** Analgesics consumption, complications, and outcome data during the postoperative period

Parameters	Group CINB (*n* = 130)	Group SI (*n* = 86)	*P*
Length of hospital stay, day	17(15–22)	16(14–20)	0.026
Postoperative hospital stay, day	8(7–10)	8(7–10)	0.033
Postoperative intubation time, h	11.0(6.5–18.0)	8.0(6.0–12.3)	0.008
Length of ICU stay, h	21.0(15.8–42.8)	22.0(18.5–43.5)	0.308
Tramadol consumption, mg	0(0–100)	100(0–225)	0.0001
Tramadol requirement	63(48%)	61(71%)	0.001
Pethidine consumption, mg	0(0–25)	25(0–50)	0.0004
Pethidine requirement	43(33%)	48(56%)	0.001
Maximal pain (VAS 0–10)	5.00(3.00–5.00)	6.00(4.75–6.00)	< 0.0001
No. of max VAS ≤ 3^a^	52(40%)	17(20%)	0.002
Dyspnea	1(0.8%)	1(1.2%)	1.000
Somnolence Pruritus	0(0%) /	1(1.2%) /	0.398
PONV	21(16%)	12(14%)	0.704
Atelectasis	4(3.1%)	2(2.3%)	1.000
Pulmonary exudation	67(52%)	49(57%)	0.433
Pleural effusion	59(45%)	41(48%)	0.741
Pneumothorax	3(2.3%)	5(5.8%)	0.270

Values are mean ± SD, median (interquartile range), or number with percentage

*Abbreviations*: *SI* Single injection, *CINB* Continuous intercostal nerve block, *ICU* Intensive care unit, *VAS* Visual analogue scale, *PONV* Postoperative nausea and vomiting

^a^Indicates that the maximal VAS score is 3 and no rescue analgesics are required

Overall, rescue analgesics were effective in all cases (reduced VAS scores; data not shown). This study found that the dose of analgesics requested by patients to relieve pain was associated with the type of intervention. The percent of patients requesting rescue drugs tramadol and pethidine in the CINB group was significantly less than that in the SI group: tramadol CINB 63 (48%) vs. SI 61 (71%), *P* = 0.001; pethidine CINB 43 (33%) vs. SI 48 (56%), *P* = 0.001. Among all patients, the drug dose of tramadol was significantly lower in the CINB group: CINB 0 (0–100) mg vs. SI 100 (0–225) mg, *P* = 0.0001; patients in the CINB group also requested lower amounts of pethidine: CINB 0 (0–25) mg vs. SI 25 (0–50) mg, *P* = 0.0004. (Table 2).

The hospital stay, postoperative hospital stay and intubation times in the CINB group were longer than those in SI, but there was no significant difference in length of ICU stay between the two groups. The incidence of PONV was about 15%, there was no skin pruritus, and only a very low incidence of untoward effects such as somnolence and dyspnea in both groups. Instances of postoperative pulmonary complications (atelectasis, pulmonary exudation, pleural effusion, and pneumothorax) were also similar between the two groups. (Table 2).

### Subgroup analysis of outcomes

#### Health care resource use

**Fig. 2:**
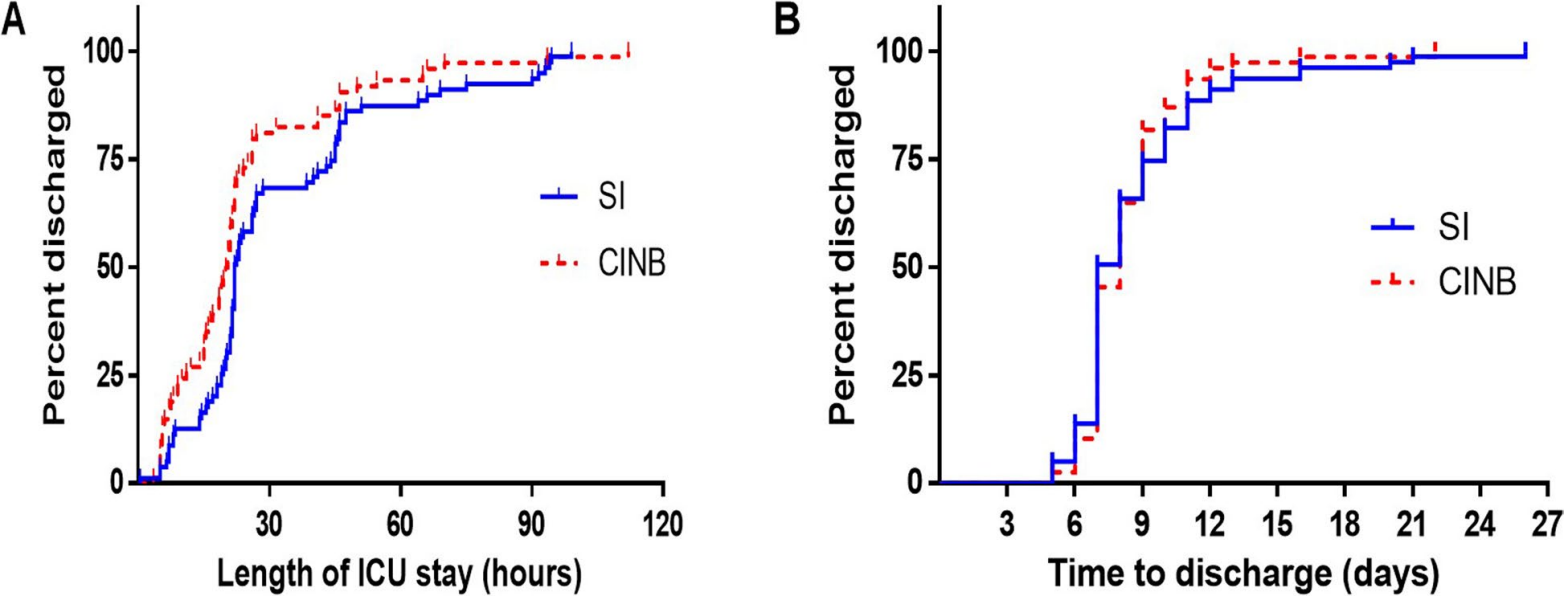
Health care resource use. Time (**A**) until discharge from intensive care unit (ICU), Log-rank (Mantel-Cox) test, *P* = 0.0241; Gehan-Breslow-Wilcoxon test, *P* = 0.0036. (B) from surgery to discharge from hospital. Log-rank (Mantel-Cox) test, *P* = 0.5858; Gehan-Breslow-Wilcoxon test, *P* = 0.8241. SI: single injection; CINB: continuous intercostal nerve block

In terms of the subgroup analysis, there was no significant difference in the baseline characteristics between the two groups, and the duration of surgery, hospital stay, postoperative hospital stay, and intubation time were also similar in both subgroups (Table S1). However, we found that the median length of ICU stay in the CINB group was 1.5 h shorter than that in the SI group (*P* = 0.011). The constructed Kaplan–Meier curves for length of postoperative ICU and hospital stays more intuitively suggest that CINB can save ICU resources when compared to SI (Table S1, Fig. 2A).

#### Pain relief and pain scores

The percentage of patients requiring rescue analgesic drugs tramadol and pethidine was significantly less in CINB than in the SI subgroup (tramadol: CINB 45% vs. SI 73%, *P* = 0.001; pethidine: CINB 31% vs. SI 58%, *P* = 0.001), which is consistent with the above comparison between the two original groups. In the CINB subgroup, the amounts of analgesics that were requested by patients to alleviate pain were also significantly lower than in the SI subgroup [tramadol: CINB 0 (0–100) mg vs. SI 100 (0–200) mg, *P* < 0.0001; pethidine: CINB 0 (0–25) mg vs. SI 25 (0–50) mg, *P* = 0.0002].

**Fig. 3:**
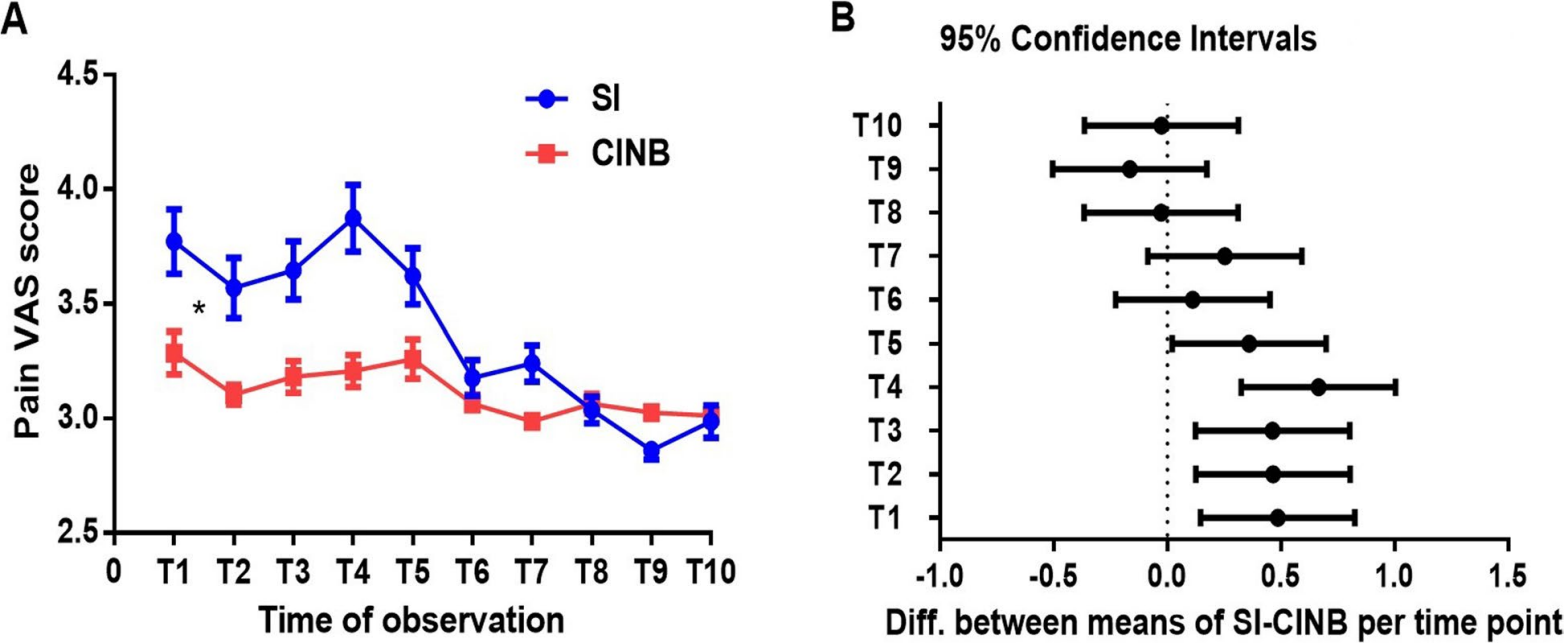
Pain trends after extubation (mean ± SEM). ANOVA with repeated measures was used to compare the pain scores between the two subgroups at successive time-points, *P < 0.0001. All of the time “points” for observation along the X-axis correspond to the time periods for pain scoring after extubation. T1: 0-2 h, T2: 4-6 h, T3: 10-12 h, T4: 20-22 h, T5: 26-28 h, T6: 34-36 h, T7: 44-46 h, T8: 50-52 h, T9: 58-60 h, T10: 68-70 h. VAS AUC (area under curve): SI = 30.41 vs. CINB = 28.05, *P* = 0.002. VAS: visual analogue scale

The maximum VAS score of 5 (3–5) in the CINB subgroup was significantly lower than that of 6 (5–6) in the SI subgroup, *P* < 0.0001 **(**Table S1). In addition, CINB resulted in a greater number of individuals achieving maximum VAS ≤ 3 (CINB 42% vs. SI 18%), *P* = 0.002. As for the area under the VAS curve in the first 70 h after extubation, this was significantly smaller in CINB: CINB 28.05 vs. SI 30.41, *P* = 0.002 (Table S1, Fig. 3A), especially the area corresponding to time periods T1-T5 (Fig. 3A, B); that is to say, CINB can alleviate pain to a significantly greater degree than SI in the first 30 h after extubation.

#### Hemodynamics/vital signs and adverse events

**Fig. 4:**
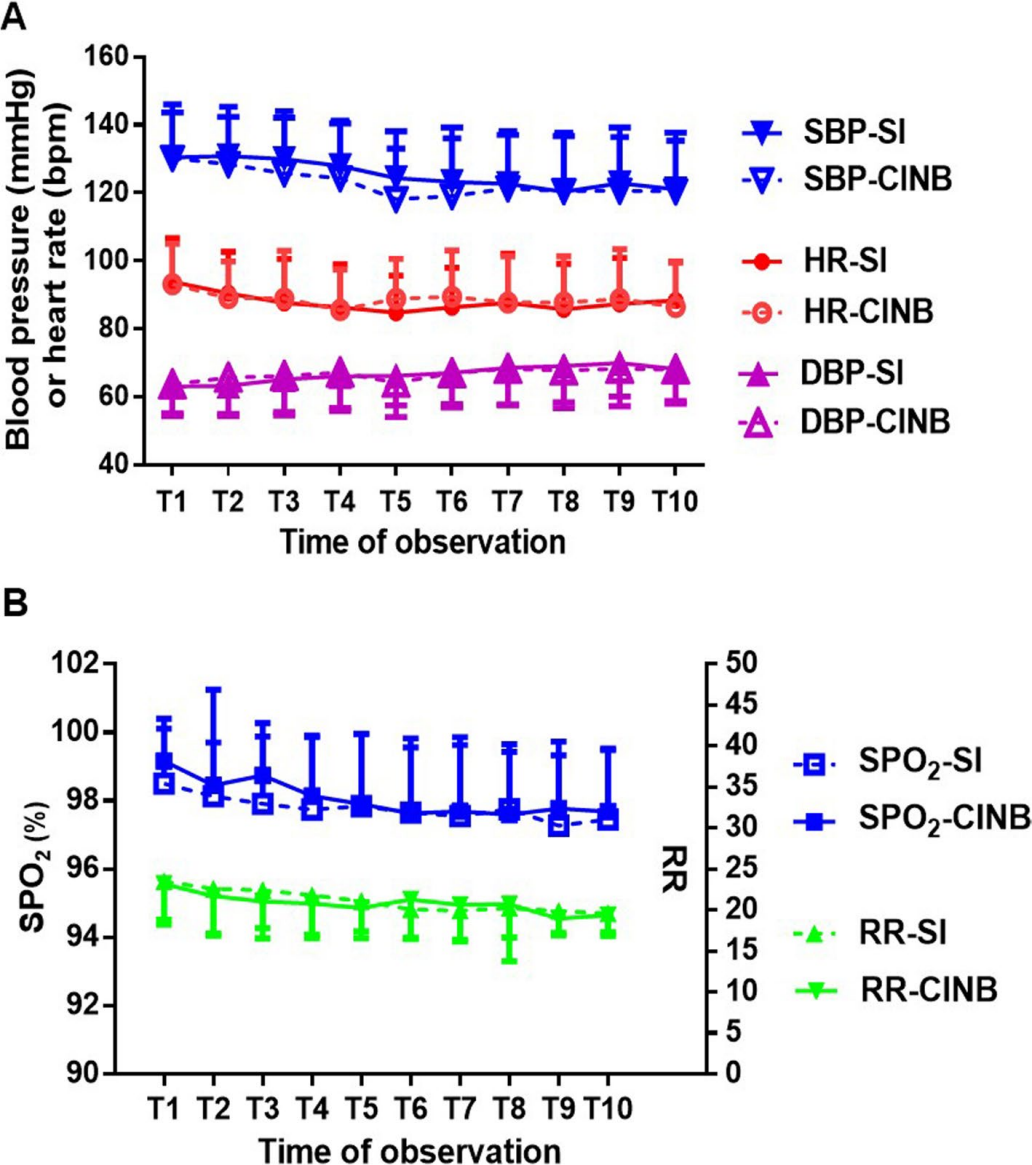
Hemodynamics/vital signs after extubation. (**A**) Hemodynamics after extubation (mean ± SD). SBP: systolic blood pressure, DBP: diastolic blood pressure, HR: heart rate; (**B**) SpO_2_ (%) or RR after extubation (mean ± SD). SPO_2_: pulse oximetry, RR: respiratory rate; SI: single injection, CINB: continuous intercostal nerve block. All of the time “points” for observation along the X-axis correspond to the time periods for pain scoring after extubation. T1: 0-2 h, T2: 4-6 h, T3: 10-12 h, T4: 20-22 h, T5: 26-28 h, T6: 34-36 h, T7: 44-46 h, T8: 50-52 h, T9: 58-60 h, T10: 68-70 h

All recorded vital signs and hemodynamic parameters, including SpO_2_, respiratory rates, systolic and diastolic blood pressure, and heart rate in 10 time periods within 70 h of extubation were stable and similar between the two subgroups (Fig. 4A, B). There was also no difference between the two subgroups in postoperative untoward effects such as PONV and pulmonary complications (Table S1).

## Discussion

Minimal invasiveness is a developmental trend of surgery – cardiac surgery is no exception [14] – and pain has always ranked among the major challenges for patients undergoing minimally invasive cardiac surgery; in particular, management of pain associated with lateral thoracotomy is of paramount importance [15–18]. The analgesic scheme for this intercostal incision has long been the focus of many scholars around the world, including epidural, paravertebral and intercostal nerve blocks, as well as intermuscular fascial block [19]. It is also reported that intercostal nerves can be blunted with frozen electrodes [5], but the resultant nerve damage is difficult to predict, and may cause hyperalgesia and chronic neuropathic pain [20]. Our institute still applies local anesthetics to infiltrate the intercostal nerves in order to help patients endure the acute perioperative pain. In our study protocol, the intercostal administration of 0.5% ropivacaine 20 ml under direct vision followed by the CINB technique (0.2% ropivacaine 5 ml/h) produced lower subjective measures of post-thoracotomy pain than for the SI group in the first 70 h after extubation.

In recent years, regional blocks have been a trend in postoperative analgesia. However, the use of epidural analgesia in cardiac surgery is limited because of hematoma caused by heparin application, and hypotension, leg weakness, and urinary retention, etc. [21] due to local anesthetic-induced sympathetic inhibition. The use of thoracic paravertebral block involves a steep learning curve, and there are also procedure-associated risks, such as pneumothorax, and concerns about the use of anticoagulant therapy. For these reasons, epidural or paravertebral blocks were not used under our institute protocol. As for the erector spinae plane block, first described in 2016, an increasing number of studies demonstrate that its effectiveness is questionable at present due to the vagaries of physical spread [22]. Some studies have reported that it has only modest analgesic effect [23, 24], and its application in MIDCAB is still controversial [25]. Therefore, the erector spinae plane block has not been applied on a large scale to date. It is reported that CINB can relieve pain in rib fracture surgery and shorten hospital stays [26], and has a superior effect in lateral thoracotomy [27]. In view of the above findings, our institute routinely applies CINB for postoperative analgesia in MIDCAB surgery. In this study, the intercostal catheter was inserted into the extrapleural space by the surgeon under direct vision [13, 28], which offers good feasibility, accurate positioning, and a reduced risk of complications. Our study shows that CINB is a superior technique to SI, offering prompt pain relief and a reductive effect on supplemental analgesics, despite the fact that more grafts were performed in this group. One study [28] has reported that the application of CINB in single port thoracoscopic surgery allowed for a reduction in pain scores, a decreased requirement for analgesics within two days after surgery, and shortened hospital stays, though the sample size is small (50 cases in each group after proportional scores matching). The differentiated point in our study relies on the fact that the intercostal incision for MIDCAB is 6 cm, and the degree of pain involved is thus much greater than that of single port thoracoscopic surgery. In this study, the analgesic effect of CINB in MIDCAB is not as good as that reported for the single port surgical model.

In terms of postoperative VAS scores and the demand for analgesics, despite CINB’s superior effect, some patients still showed signs of insufficient analgesia, which may be attributed to the following reasons: 1) The catheter for continuous intercostal nerve block was not placed in the optimal position by the surgeon, or was displaced after surgery; 2) CINB only acts on a single intercostal space, and the pain between adjacent intercostals due to the application of surgical retractors is not suppressed; 3) individual discrepancies in pain sensation, or the accidental rupture of intercostal nerves caused by surgical incision may cause a few individuals to experience either no pain or hyperalgesia.

The area under the VAS curve in the first 70 h after extubation for CINB was significantly smaller than for SI (Fig. 3A), especially in T1-T5 (Fig. 3A, B), i.e., the difference between the two groups was most obvious in the first 30 h after extubation, which may be related to the effective period of the continuous intercostal nerve block. It is a predictable phenomenon that the SI effect has completely worn off by T1 since the median extubation time for the SI group is 8 h. Considering that the pain levels of both groups decreased 30 h after extubation, and that the median postoperative intubation time was about 9 h, it can be inferred that the characteristic of pain management in MIDCAB is that the degree of pain will significantly reduce two days postoperatively after intercostal nerve block (single or continuous), which is consistent with the finding that the reported pain degree of lateral thoracotomy in MIDCAB [25] is generally most severe within 2–3 days after surgery.

High-dose opioids administered within a short period of time will negatively affect blood pressure, heart rate and respiration; in particular, the effective ventilation of patients will drastically worsen with the increased sedation [29]. The application of regional nerve block to relieve pain can decrease stress response, reduce the side effects of opioids in favor of coughing and ventilatory efforts, and facilitate oxygen supply to the myocardium [8, 30]. In our study, due to the administration of intercostal nerve block (single or continuous), we observed few side effects such as somnolence, PONV, respiratory depression, and, in rare cases, atelectasis; at the same time, both CINB and SI analgesia protocols presented with stable hemodynamic conditions and good respiratory parameters.

There are reports in the literature of cases of pulmonary exudation and pleural effusion following thoracotomy, which are considered to be related to internal mammary artery harvesting [31] and lung collapse due to one lung ventilation [32]. Despite a low incidence of postoperative atelectasis and pneumothorax in both groups of the present study, it is worth mentioning that the rates of pulmonary exudation and pleural effusion in each group were still around 50%, suggesting that although it is MIDCAB surgery, the lung protection strategy for left intercostal thoracotomy with internal mammary artery harvesting and one lung ventilation should be improved. On this issue, further exploration and research are currently underway in our group.

Indeed, the analysis of the original groups showed that although CINB fared significantly better than SI in terms of pain relief, it was longer in terms of hospital stay and postoperative intubation time, and there was no significant difference in length of ICU stay between the two groups. We believe this may be attributed to the fact that there were more patients with No. grafts ≥ 2 in the CINB group, indicating a weaker overall physiological condition among this group of patients, which would tend to be accompanied by longer duration of surgery and prolonged postoperative monitoring times. Subgroup data analysis showed that CINB still had superior analgesic effect and could shorten the length of ICU stay, though there was no difference in hospital stay or postoperative intubation time between the two groups. It is also worth pointing out that hospital stays are long because our institution has integrated cardiovascular medicine and cardiac surgery, and because the hospital stay prior to surgery was also included in the total hospital stay.

In addition to the benefits in postoperative analgesia, the single graft subgroup analysis mentioned above also showed that ICU stays for the CINB group were significantly shortened compared with the SI group. Therefore, intercostal nerve block also has a positive impact on the utilization and allocation of medical resources. Some scholars reported in a small sample size study [30] that single intercostal nerve block can promote early extubation, shorten the duration of ICU stays and relieve postoperative pain in minimally invasive mitral valve surgery, which is consistent with the results of our study. Generally speaking, while the duration of ICU and hospital stays may be associated with the protocols of physicians in arranging discharge, they are also obviously affected by postoperative organ function and nursing requirements [33]. For single graft coronary artery bypass surgery, there was no difference in the length of hospital stay between the two groups; however, we found that the ICU stay in the CINB group was significantly shorter, which was also suggested by the Kaplan–Meier curves analysis. It is our view that the lesser requirements for supplemental analgesics in the CINB group may have led to shorter nursing times in ICU. Why, then, is there a similar postoperative intubation time between the two groups, but a difference in ICU stays? This is because patients with tracheal intubation are under sedation. Generally, supplemental analgesic drugs were administered according to VAS score, which was evaluated after extubation. Therefore, the advantage of CINB became apparent only after extubation.

### Limitations

1. This study is limited by the short period of postoperative observation and follow-up. The results of this study reflect the improvement of pain in the initial 70 h after extubation; the impact on long-term chronic pain control or overall recovery is unclear. However, effective initial pain control is an important factor in patient recovery and satisfaction.
2. This study is a retrospective study. The PCA pump pressing used in the study was nurse assisted. The noted effective pressing times in the medical records were not collected, although this did not affect the results of the study.
3. This is a real-world retrospective study, focusing on actual analgesic effects. Therefore, only the evaluation of pain degree after extubation is considered. The dosage of drugs during anesthesia and sedation were not collected in this study, and these were considered to have been metabolized by the time of extubation.

## Conclusions

The results of this retrospective cohort study indicate that CINB provided better analgesia with a reduced length of ICU stay compared to that obtained by a single injection alone in patients undergoing MIDCAB. Nevertheless, its effect on some patients is not ideal. We further recommend to conduct RCT trial studies, exploring better analgesic schemes such as intermittent bolus regimens and poly-points blocks, to improve CINB performance in postoperative pain control and increase the benefits to patients.

## Supplementary Information

**Additional file 1.**

## Data Availability

The datasets used and/or analyzed during the current study are available from the corresponding author on reasonable request.

## Abbreviations

MIDCAB: Minimally invasive direct coronary artery bypass
CINB: Continuous intercostal nerve block
SI: Single block
VAS: Visual analogue scale
RCT: Randomized controlled trial
IABP: Intra-aortic balloon pump
ICU: Intensive care unit
CCU: Cardiac care unit
PCA: Patient-controlled analgesia
PONV: Postoperative nausea and vomiting
LIMA: Left internal mammary artery
