# Supplementary material for: The effect of continuous intercostal nerve block vs. single shot on analgesic outcomes and hospital stays in minimally invasive direct coronary artery bypass surgery: a retrospective cohort study
Source: BMC Anesthesiol. 2022 Mar 8;22:64. doi: 10.1186/s12871-022-01607-7 (PMC8903669; doi:10.1186/s12871-022-01607-7)
Supplement: Supplementary file 1 — Additional file 1. [file 12871_2022_1607_MOESM1_ESM.docx]

**Table S1. Subgroup analysis (No. of grafts =1) of the relevant data.**

| **Parameters** | **Group CINB (n=77)** | | **Group SI (n=79)** | ***P*** |
| --- | --- | --- | --- | --- |
| **Age, years** | 62.1±10.3 | 62.4±10.8 | | 0.870 |
| **Weight, kg** | 69.1±12.2 | 69.5±9.9 | | 0.807 |
| **Male, %** | 57(74%) | 53(67%) | | 0.383 |
| **BMI, kg.m^-2^** | 25.1±3.5 | 25.1±2.9 | | 0.940 |
| **Hypertension** | 53 (69%) | 51 (65%) | | 0.613 |
| **Diabetes** | 26 (34%) | 25 (32%) | | 0.865 |
| **NYHA** |  |  | | 0.656 |
| **I/asymptomatic** | 22(29%) | 27(34%) | |  |
| **II** | 41(53%) | 41(52%) | |  |
| **III** | 14(18%) | 10(13%) | |  |
| **IV** | 0(0%) | 1(1%) | |  |
| **Pulmonary function *** |  |  | |  |
| **FVC actual, L** | 3.17±0.77 | 3.10±0.86 | | 0.633 |
| **FVC % predicted** | 87±14 | 85±15 | | 0.468 |
| **FEV_1_ actual, L** | 2.37±0.68 | 2.45±0.68 | | 0.528 |
| **FEV_1_ % predicted** | 82±17 | 86±16 | | 0.253 |
| **Pre. PO_2_, mmHg^#^** | 80±15 | 81±14 | | 0.508 |
| **Pre. PCO_2_, mmHg ^#^** | 39±3 | 40±4 | | 0.436 |
| **Pre. glucose, mmol/L** | 7.8(5.8-9.4) | 7.0(5.8-9.0) | | 0.294 |
| **Duration of surgery, h** | 2.3(1.9-2.7) | 2.5(2.1-2.9) | | 0.099 |
| **Length of hospital stay, day** | 16(14-22) | 15(13-20) | | 0.329 |
| **Postoperative hospital stay, day** | 8(7-9) | 7(7-10) | | 0.825 |
| **Postoperative intubation time, h** | 9.0(6.0-15.0) | 7.5(6.0-12.8) | | 0.395 |
| **Length of ICU stay, h** | 20.5(11.3-26.0) | 22.0(19.0-45.0) | | 0.011 |
| **Tramadol consumption, mg** | 0(0-100) | 100(0-200) | | <0.0001 |
| **Tramadol requirement** | 35(45%) | 58(73%) | | 0.001 |
| **Pethidine consumption, mg** | 0(0-25) | 25(0-50) | | 0.0002 |
| **Pethidine requirement** | 24(31%) | 46(58%) | | 0.001 |
| **Maximal pain (VAS 0–10)** | 5.00(3.00-5.00) | 6.00(5.00-6.00) | | <0.0001 |
| **No. of max VAS≤3†** | 32(42%) | 14(18%) | | 0.002 |
| **AUC of VAS** | 28.05 | 30.41 | | 0.002 |
| **dyspnea** | 1(1.3%) | 1(1.3%) | | 1.000 |
| **Somnolence** | 0(0%) | 1(1.3%) | | 1.000 |
| **PONV** | 16(21%) | 11(14%) | | 0.294 |
| **Atelectasis** | 4(5.2%) | 2(2.6%) | | 0.439 |
| **Pulmonary exudation** | 44(57%) | 47(59%) | | 0.871 |
| **Pleural effusion** | 41(53%) | 37(47%) | | 0.522 |
| **Pneumothorax** | 0(0%) | 4(5.1%) | | 0.120 |

NOTE. Values are mean ± SD, median (interquartile range), or number with percentage. Abbreviations: SI, single injection; CINB, continuous intercostal nerve block; NYHA, New York Heart Association; FEV1, forced expiratory volume in 1 second; FVC, functional vital capacity; BMI, body mass index; Pre., preoperative; ICU, intensive care unit; VAS, visual analogue scale; AUC, area under curve; PONV, postoperative nausea and vomiting. *indicates that only 44 patients in CINB group had preoperative pulmonary function data. # Indicates n=75 in SI, and n=74 in CINB. † Indicates that the maximal VAS score is 3 and no rescue analgesics are required.
